# Traumatic dental injuries among children attending the public after-hours emergency dental clinic in Bergen, Norway

**DOI:** 10.2340/aos.v83.40622

**Published:** 2024-05-14

**Authors:** Faiza D. Sælen, Jorma I. Virtanen, Marit S. Skeie, Gerhard Sulo, Dorina S. Thelen

**Affiliations:** a Department of Clinical Dentistry, Faculty of Medicine, University of Bergen, Bergen, Norway; b Oral Health Center of Expertise in Western Norway, Bergen, Norway; c Institute of Dentistry, University of Turku, Turku, Finland; d Center for Oral Health Services and Research, Mid-Norway (TkMidt), Trondheim, Norway; e Department of Global Public Health and Primary Care, Faculty of Medicine, University of Bergen, Bergen, Norway

**Keywords:** Trauma, epidemiology, dentition, permanent, emergencies

## Abstract

**Objectives:**

To investigate traumatic dental injuries (TDIs) among children who for 1 year attended a Norwegian public after-hours emergency public dental (EPD) clinic.

**Materials and methods:**

The study included 7–18-year-olds (*n* = 312) who presented at the EPD clinic, underwent a clinical dental examination, and consented to the disclosure of clinical information. Recording of TDIs was restricted to anterior permanent teeth. Potential TDI predictors were also analysed.

**Results:**

Almost half (*n* = 148) of the children were assessed with TDIs in permanent teeth, showing a mean age of 11.0 (standard deviation [SD]: 3.5) years. Males constituted 54.7%. The children experienced TDIs often outside school hours (43.9%), and the majority (58.1%) were caused by falls/accidents. Sixty of them experienced only one TDI. The most common location was the maxillary central incisors. Assessment of TDIs according to severity, could only be done in 131 individuals, involving 253 TDIs. Of these, 81.8% were mild. The odds of visiting the emergency clinic for a TDI were higher (odds ratio [OR] = 2.64, confidence interval [CI]: 1.61–4.31) among children with previous TDIs and lower (OR = 0.28, CI: 0.12–0.68) among those with poor dental attendance.

**Conclusions:**

Traumatic dental injuries were a common reason for seeking emergency care. Milder injuries dominated and involved mostly one maxillary central incisor. Previous episodes of TDIs and attendance patterns seemed to be associated with seeking care for TDIs.

## Introduction

Traumatic dental injuries (TDIs) put a substantial burden on the public health care system [1], and have long-term consequences for oral health [2]. TDIs among children, adolescents and young adults occur suddenly and require urgent attention [3]. Yet, they are often overlooked in surveys, due to the fact that they are inconsequential compared with other forms of injuries [4]. More than 1 billion people have experienced TDIs during their lifetime [5]. Their prevalence varies widely, from 6 to 59% [2, 3]. This variation is attributable to many factors, including – but not confined to – differences in the demographics of populations studied, TDIs classification systems used, and reporting modalities [3]. In Norway, the prevalence of TDIs among 16-year -olds is reported to be 6.4%, with mild cases dominating the clinical picture [6], while the incidence is reported to vary from 1.3 to 2.0% [7].

Studies presenting children with TDIs at after-hours emergency clinics [8–12] suggest that such cases are more severe and involve more teeth compared to trauma occurring during ordinary working hours [8, 9]. There is also evidence of major delays [13], often exceeding 24 h [14] in treatment. From this perspective, the availability and quality of emergency services at after-hours clinics are very important as optimal emergency management might be crucial for the TDI prognosis. The importance of immediate attention for the handling of TDIs, is gradually being recognised by the guardians of children [15].

To our knowledge, no previous publications have addressed TDIs in children attending after-hours clinics in Norway. Therefore, we aimed to quantify the number and characteristics of children who due to TDIs attended a Norwegian after-hours emergency clinic, the number of teeth involved by TDIs, and how the TDIs were distributed in the dentition. Additionally, we aimed to assess the impact of background factors on TDIs.

## Materials and methods

### Study participants

**Figure 1:**
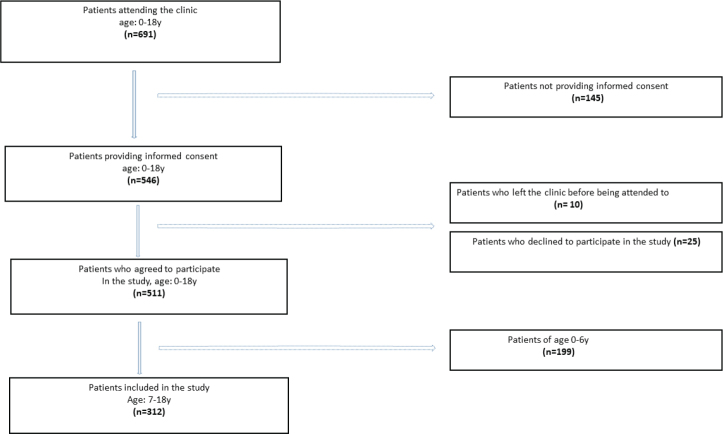
Flow diagram of study patients.

In resource-rich countries such as the Scandinavian countries (Norway, Sweden, and Denmark), all children are given free-of-charge dental care by the Public Dental Service (PDS). Nevertheless, public after-hours emergency clinics coexist with the PDS clinics which are open on working days only, to ensure that all children in need of urgent dental assistance, are taken care of. The background for the present study was to focus on these children to get an impression of all available TDIs taking place outside ordinary working hours. The study therefore was conducted in the Emergency Public Dental (EPD) clinic, located in Bergen, Norway. The EPD clinic collaborates with the ordinary PDS clinics and provides emergency care after working hours (weekdays 18.00–20.30; and on weekends/public holidays 15.30–20.30). The present study was part of a more extensive project, describing the characteristics of 691 children and adolescents ≤ 18 years presenting at the EPD clinic [16]. Of these, 145 did not give informed consent, 10 left the clinic before being seen by dental health personnel, and 25 declined to participate. Of the 511 remaining children, 199 were aged 0–6 years. As the current study focused on TDIs occurring in permanent teeth only, the remaining study sample constituted 312 children, 7–18 years old (Figure 1).

### Assessment of TDIs

In a pilot study conducted in 2017, dental health personnel were trained in how to approach eligible children and were calibrated in assessing TDIs and their treatment options. The present register-based study reported on TDIs involving anterior permanent teeth (canine to canine, in both maxilla and mandible). Information on TDIs was obtained from the participant’s Electronic Patient Journal (EPJ) and was based on the World Health Organization (WHO) criteria modified by Glendor et al. [17]. TDIs have been routinely registered in EPJ (Opus Dental) since 1998. Based on clinical severity, TDIs were classified as ‘*mild*’, ‘*moderate*’ or ‘*severe*’. *Mild* injuries were defined as enamel infraction, enamel fracture, enamel/dentin fracture, concussion, or subluxation (horizontal mobility). *Moderate* injuries were defined as complicated crown fracture (pulp involved), uncomplicated crown-root fracture, root fractures in the apical or middle third without luxation of the coronal fragment or subluxation (vertical mobility). *Severe* injuries were defined as complicated crown-root fracture (pulp involved), root fracture in the cervical one-third, root fracture in the middle or apical third with luxation of the coronal fragment, extrusive luxation, lateral luxation, intrusive luxation or avulsion (exarticulation). The examiners (*n* = 9) were experienced dentists, employed at EPD clinic for years (15.2 (SD: 10.6) years). Their mean age was 51.7 (SD: 9.3) years.

### Additional information

Additional information relevant to our study included the participant’s age, gender, the time and circumstances in which the TDIs occurred, and dental attendance pattern in ordinary PDS. Seasonality of the TDIs was categorised into ‘winter’ (December–February), ‘spring’ (March–May), ‘summer’ (June–August), and ‘autumn’ (September–November). ‘Average attendance’ was defined as occasionally (up to twice) failing to keep a scheduled dental appointment without notice; ‘poor attendance’ was defined as frequently (≥ 3 times) failing to keep a scheduled dental appointment without notice.

### Statistical analysis

Data were analysed using Stata Statistical Software (College Station, Texas, USA: Stata Corp LP) STATA, version 16. Continuous variables are expressed as mean and SD or median (interquartile range [IQR]) – depending on the distributional pattern. Categorical variables are expressed as the number (proportion) of responders. The association between variables of interest was tested using the chi-square or Fisher’s exact test for categorical variables and independent-samples *t*-test for continuous variables. Logistic regression models were used to explore the association between gender, age, seasonality of TDIs, previous TDIs, dental attendance pattern and seeking care for a current TDI. The results are expressed as OR and corresponding 95% CI. Two-sided tests with the 0.05 significance level were used.

### Ethical approval

Ethical approval for this study was obtained from the relevant Social Science Data Services (NO.52504). Study approval was also granted from the chief of the EPD clinic and the head of the PDS, Hordaland County. The patients or their parents agreed to participate in the study and consented to our accessing their EPJ for relevant information. Caregivers were involved if participants were under 16 years.

## Results

### The participants

The characteristics of the study participants are summarised in Table 1. Their mean age was 10.9 (SD: 3.4) years and 54.7% were males. They were mostly (95.2%) residents of Hordaland.

**Table 1:** Baseline characteristics of the patients (*n* = 312) seeking care at the EPD clinic.

Characteristics	Total (*n* = 312)						TDI (*n* = 148)						Non TDI (*n* = 164)					
	*n*	%	Mean	SD	Median	IQR	*n*	%	Mean	SD	Median	IQR	*n*	%	Mean	SD	Median	IQR
Sex, *n* (%)																		
Male	169	54.7					81	54.7					88	53.7				
Female	143	45.3					67	45.3					76	46.3				
Resident in, *n* (%)																		
Hordaland	297	95.2					141	95.3					156	95.1				
Other	15	4.7					7	4.7					8	4.9				
Age (in years)																		
Mean (SD)			10.9	3.4					11.0	3.5					10.8	3.3		
Median (IQR)					10.0	8–13					10.0	3.5					10	8–13
Season, *n* (%)																		
Winter	68	21.8					35	23.7					33	20.1				
Spring	70	22.4					40	27.0					30	18.3				
Summer	94	30.2					40	27.0					54	32.9				
Autumn	80	25.6					33	22.3					47	28.7				

TDI: traumatic dental injury; SD: standard deviation; IQR: Interquartile range; EPD: Emergency Public Dental.

For 148 individuals (47.4%), a TDI was the reason for visiting the EPD clinic. Participants with TDIs accounted for 68.2% of all visits to the EPD in November and 31.0% in September and October, respectively. The proportion of individuals (within total) was high in February, tended to decline throughout the summer months, and rose again sharply in November. In males, the number of children peaked in February and in November while in females, TDIs peaked in July.

Most children experienced TDIs outside school hours (43.9%), and their TDIs were accidental and involved a fall (58.1%) (Table 2), mostly at home or during cycling and training activities (either organised or private).

**Table 2:** Location and causes of TDIs.

	*n*	%
Where trauma occurred		
At school (during school hours)	19	12.9
Outside school (not during school hours)	65	43.9
At home	28	18.9
Not reported/missing	36	24.3
Cause of TDIs		
Fall/accident	86	58.1
Violence	2	1.4
Sport-related	14	9.5
Recreational time (jumping, playground)	8	5.4
Cycling	11	7.4
Other	18	12.1
Not reported/missing	9	6.1

TDI: traumatic dental injury.

**Figure 2:**
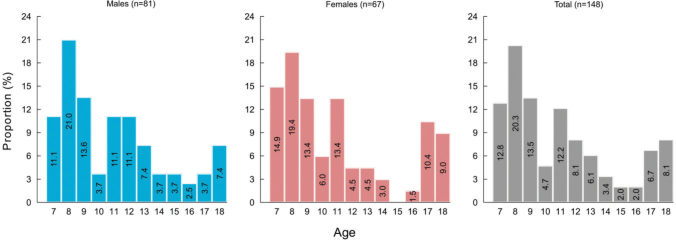
Age distribution of study patients: overall and by sex.

Figure 2 summarises the overall and sex-specific age distribution of children presenting at the EPD clinic with TDIs. The mean (SD) age at trauma was 11 (3.5) years, with 8-year-olds accounting for 20.3% of the TDIs episodes and negligible difference between males (11 [SD: 3.4] years) and females (11 [SD: 3.7] years). The 8-year-olds accounted for 21.0 and 19.4% of all TDIs in males and females, respectively.

### The TDIs and distribution

For 17 participants (11.4% of all children with TDIs), the severity of TDIs could not be determined because information about the clinical diagnosis of the involved tooth or teeth was unavailable. Classification according to severity in 131 participants thus involved 253 TDIs (Table 3). Of these TDIs, 81.8% (*n* = 207) were mild, 7.1% (*n* = 18) were moderate, and 11.1% (*n* = 28) were severe.

**Table 3:** Distribution of TDIs amongst the patients (*n* = 131), stratified by severity.*

Diagnosis	Severity	Number
Injuries to the hard dental tissues and the pulp (*n* = 114)		
Enamel infraction	Mild	4
Enamel fracture	Mild	23
Enamel-dentin fracture	Mild	68
Complicated crown fracture	Moderate	11
Uncomplicated crown-root fracture	Moderate	3
Root fracture	Moderate	4
Complicated crown fracture	Severe	1
Injuries to the periodontal tissues (*n* = 139)		
Concussion	Mild	92
Subluxation with horizontal mobility	Mild	20
Subluxation with vertical mobility	Severe	7
Extrusive luxation	Severe	6
Lateral luxaion	Severe	11
Intrusive luxaion	Severe	1
Avulsion	Severe	2
Total		253

TDI: traumatic dental injury.

* 253 traumatised teeth are included in these analyses.

The maxillary central incisors were the most frequently injured, followed by the maxillary lateral incisors and the mandibular central incisors. Clinically, mild injuries predominated in all locations.

### Distribution of TDIs among children involving single or multiple teeth.

In 60 patients, only one tooth was traumatised, most frequently a maxillary central incisor (88,3%). Of those 60 patients, 53.3% experienced TDIs on tooth 11 and 35.0% on tooth 21, followed by the maxillary left lateral incisor (36.6%). In 37 children, TDIs involved two teeth; most frequently both maxillary central incisors (75.6%), and maxillary central and lateral incisors. In 17 children, the TDIs involved three teeth. Maxillary central incisors and lateral incisors (35.3%) were most often involved. Moreover, there were also 16 children involving multiple TDIs on anterior teeth: up to four (*n* = 13), five (*n* = 2), or even six (*n* = 1) teeth.

### Background factors

No association was observed between the study outcome (i.e., attending the EPD clinic because of a TDI) and the participants’ age, gender or seasonality for TDIs. However, the odds of presenting at the clinic with a TDI were higher (OR = 2.64, CI: 1.61–4.31) among children with a previous TDI. The opposite pattern was observed among patients with ‘poor’ dental attendance pattern in the PDS compared to patients with regular attendance habits; they visited the emergency clinic less frequently for a TDI (OR = 0.28, CI: 0.12–0.68) (Table 4).

**Table 4:** Association between characteristics of the patients presenting at the EPD clinics and TDIs.

Characteristics	Odds ratio (95% CI)
Sex	
Female	1 ^ref^
Male	1.04 (0.67–1.63)
Age *(1 year increase)*	1.02 (0.95–1.09)
Season	
Winter	1 ^ref^
Spring	1.26 (0.64–2.46)
Summer	0.70 (0.37–1.31)
Autumn	0.66 (0.35–1.27)
Attendance pattern at PHDS clinics	
Regular	1 ^ref^
Average	0.62 (0.28–1.34)
Poor	0.28 (0.12–0.68)
Previous TDI	
No	1 ^ref^
Yes	2.64 (1.61–4.31)

PHDS: public dental service; TDI: traumatic dental injury; EPD: Emergency Public Dental.

## Discussion

To the best of our knowledge, this is the first study in Norway to quantify the number and characteristics of children attending an after-hours EPD, the number of TDIs involved, and their distributional patterns. Children with TDIs accounted for nearly half (47.4%) of all visits. No significant differences were observed with respect to the children’s age, gender, or season of TDIs. Among children with a TDI, age was disproportionally distributed, with TDIs peaking in the 8th year of life. The children experienced TDIs more often outside school hours, and were mainly caused by falls/accidents during activities (both unorganised and organised). TDIs involved mainly one or two teeth, occurred most often in the maxillary anterior teeth, and were usually classified as mild (81.8%).

These findings cannot be compared directly with the existing literature on the topic, because of differences in the structure and function of dental healthcare systems, study periods, age and gender distribution of study participants, and geographical variation. The present findings are however, in line with two international studies in which TDIs represented the most frequent reason for attending emergency clinics 51 [18] and 46% [19], respectively. In contrast, TDIs accounted for only 5% of visits in another study conducted at a dental school emergency clinic in Brazil [20]. The authors of the study attributed this to the fact that school attendees had access to a specialised dental trauma care service, to which most trauma-affected children were immediately referred.

The peak age for TDIs in our study was 8 years, similar to that reported in international [4, 14] and other Norwegian [7] studies, reflecting the vulnerability to TDIs at this age. However, the proportion of males (54.7%) was lower than that reported in a review of literature by Bastone et al. [21] (56–70%), and another individual study conducted in a similar setting in Newcastle, Australia (72%) [8]. Other studies of TDIs treated at after-hours clinics also report a higher proportion of males than females [9, 12]. The observed increase in the proportion of females experiencing TDIs in our study may reflect the increasing proportion of girls engaged in recreational activities in Norway.

The findings of the present study are also in accordance with previous studies [2, 22] describing clinical cases of TDIs as predominantly uncomplicated, mild injuries. However, our proportion of severe TDIs (11.1%) was lower than in Warren et al. study (26.0%) including avulsions and luxations [12], but lower than in two other Norwegian studies (4.0 and 5.5%, respectively) [6, 22]. Other published studies suggest that luxation and avulsion injuries (severe TDIs) are more common in after-hours emergency services [9, 23]. The higher proportion of severe TDIs reported in after-hours studies (compared to ‘normal’ hours studies) may reflect the fact that children experiencing minor, mild injuries may wait until the next day(s) and visit clinics during working hours.

Most of the children in our study experienced that their TDIs were of accidental origin and occurred during cycling and sports activities, which is in line with literature [24]. Our findings are also in accordance with previous reports indicating falls [10] and random accidents [12] as the main reasons for TDIs.

There is consensus in the published literature that the main type of a TDI involves injury to the periodontal tissues, with or without concomitant injuries to the hard tissues, including root fracture (73%) and that the most frequently affected teeth are the maxillary central incisors [1, 2, 6, 9, 12].

Some studies have reported an increase in TDIs during the winter, while others report an increase during the summer months [20]. In our study, there was no clear seasonal trend when patients experienced TDI. However, the differences between boys and girls might be related to differences in indoor and outdoor activities between the genders.

The importance of immediate attention for the handling of TDIs has been emphasised [13, 15]. We observed that a considerable proportion of our study population reported previous TDIs. This is in accordance with published literature [2], and may indicate the need for more extensive research with a focus on this high-risk subgroup of children.

### Strengths and limitations

The research of TDIs among children after working hours is scarce and this study adds to this gap. Our study was conducted in Bergen, which has only one, central after-hours EPD clinic, thus serving as a catchment area for the whole Hordaland County. One of the strengths of this study is the use of the EJS to link information from the current visit with that from the PDS. The standardised EPJ format including the standardised TDI form, ensured reliable and valid clinical data (historical data from EPJ). Another strength was that the study was conducted over a whole year, making it possible to explore potential changes in attendance trends in relation to the various seasons.

The main limitation was the considerable number of eligible patients who did not (for practical reasons) participate in the study. Furthermore, some TDIs may have been treated by private dental practitioners.

## Conclusions

Traumatic dental injuries were a common reason for seeking care. The children’s TDIs were accidental, often occurring after school hours. Milder injuries dominated and involved most often one maxillary central incisor. Previously experienced TDIs were associated with higher odds of seeking care due to a TDI, while a poor attendance pattern in PDS was associated with lower odds. Most often, episodes of trauma involved 8-year-olds and boys.
